# MCPIP1 modulates the miRNA‒mRNA landscape in keratinocyte carcinomas

**DOI:** 10.1186/s13046-024-03211-8

**Published:** 2024-10-21

**Authors:** Agata Lichawska-Cieslar, Weronika Szukala, Guillem Ylla, Gabriela Machaj, Faustyna Ploskonka, Iwona Chlebicka, Jacek C. Szepietowski, Jolanta Jura

**Affiliations:** 1 Faculty of Biochemistry, Biophysics and Biotechnology, Department of General Biochemistry, Jagiellonian University, Gronostajowa 7, Krakow, 30-387 Poland; 2 Doctoral School of Exact and Natural Sciences, Jagiellonian University, Lojasiewicza 11, Krakow, 30- 348 Poland; 3 Faculty of Biochemistry, Biophysics and Biotechnology, Laboratory of Bioinformatics and Genome Biology, Jagiellonian University, Gronostajowa 7, Krakow, 30-387 Poland; 4 Department of Dermatology, Venereology and Allergology, Wroclaw Medical University, Chalubinskiego 1, Wroclaw, 50-368 Poland; 5 Faculty of Medicine, Wroclaw University of Science and Technology, Grunwaldzki sq. 11, Wroclaw, 51-377 Polska

**Keywords:** MCPIP1, Regnase-1, SCC, Skin cancer, Keratinocytes, miRNA, Transcriptomics

## Abstract

**Background:**

Monocyte Chemotactic Protein 1-Induced Protein 1 (MCPIP1, also called Regnase-1) is a negative modulator of inflammation with tumor-suppressive properties. Mice with keratinocyte-specific deletion of the *Zc3h12a* gene, encoding MCPIP1, (Mcpip1^eKO^ mice) are more susceptible to the development of epidermal papillomas initiated by 7,12-dimethylbenz[a]-anthracene (DMBA) and promoted by 2-O-tetradecanoylphorbol-13-acetate (TPA).

**Methods:**

The aim of this study was to investigate the MCPIP1 RNase-dependent microRNA (miRNA)‒mRNA regulatory network in chemically induced squamous cell carcinoma (SCC)-like skin papillomas. Next-generation sequencing (NGS) coupled with bioinformatic analysis was used to shortlist the MCPIP1-dependent changes in protein-coding genes and miRNAs. The expression levels of the selected miRNAs were analyzed by quantitative PCR in human keratinocytes with MCPIP1 silencing. Functional studies were performed in human keratinocytes transfected with appropriate miRNA mimics. The DIANA-microT-CDS algorithm and DIANA-TarBase v7 database were used to predict potential target genes and identify the experimentally validated targets of differentially expressed (DE) miRNAs.

**Results:**

RNA sequencing (RNA-Seq) analysis of control and Mcpip1^eKO^ DMBA/TPA-induced papillomas revealed transcriptome changes, with 2400 DE protein-coding genes and 33 DE miRNAs. The expression of miR-223-3p, miR-376c-3p, and miR-139-5p was confirmed to be dependent on MCPIP1 activity in both murine and human models. We showed that MCPIP1 directly regulates the expression of miR-376c-3p *via* direct cleavage of the corresponding precursor miRNA. The pro-proliferative activity of miR-223-3p, miR-376c-3p, and miR-139-5p was experimentally confirmed in SCC-like keratinocytes. Bioinformatic prediction of the mRNA targets of the DE-miRNAs revealed 416 genes as putative targets of the 18 upregulated miRNAs and 425 genes as putative targets of the 15 downregulated miRNAs. Further analyses revealed the murine interactions that are conserved in humans. Functional analysis indicated that during the development of cutaneous SCC, the most important pathways/processes mediated by the miRNA‒mRNA MCPIP1-dependent network are the regulation of inflammatory processes, epithelial cell proliferation, Wnt signaling, and miRNA transcription.

**Conclusions:**

Loss of MCPIP1 modulates the expression profiles of 33 miRNAs in chemically induced Mcpip1^eKO^ papillomas, and these changes directly affect the miRNA‒mRNA network and the modulation of pathways and processes related to carcinogenesis.

**Supplementary Information:**

The online version contains supplementary material available at 10.1186/s13046-024-03211-8.

## Introduction

Skin cancers are among the most common cancers worldwide. In general, they are of two types: melanoma and nonmelanoma skin cancers. Nonmelanoma skin cancers (NMSCs) are caused by genetic and environmental factors and account for approximately 95% of skin cancers (1,2). Among this type of cancers, most are basal cell carcinomas (BCCs; 70%) or squamous cell carcinomas (SCCs; 25%) [1–3]. Cutaneous SCC (cSCC) has one of the highest mutation rates among human cancers. In addition, it is more aggressive and metastasizes to lymph nodes faster than BCC [4]. Skin carcinogenesis involves complex interactions between the immune system and developing tumors. Dysregulations in innate immunity, including altered expression of toll-like receptors (TLRs), antimicrobial peptides, and proinflammatory cytokines (e.g. IL-1α), play a critical role in skin carcinogenesis [5, 6]. These mechanisms involve the coordinated action of several RNA-binding proteins responsible for mRNA decay. This regulation is essential to prevent the activation of an aberrant immune response. Inflammation, especially chronic one, is the definite cause for tumor development and progression [7].

Recently, we established a mouse model in which the developing skin papillomas had early features of cSCC [8]. In the model mice, the *Zc3h12a* gene encoding Monocyte chemotactic protein 1-induced protein 1 (MCPIP1), also called Regnase-1, was specifically knocked out in keratinocytes (Mcpip1^eKO^ (epidermal knockout) mice). MCPIP1 is an RNase possessing a PilT N-terminus (PIN) domain with catalytic properties [9, 10]. As this protein is strongly activated by proinflammatory cytokines and negatively regulates proinflammatory cytokine levels, it was initially described as an important anti-inflammatory factor [9–12]. However, further studies have shown that MCPIP1 is also involved in the regulation of processes such as cell division, angiogenesis, differentiation, macrophage polarization and apoptosis [13–16]. In addition to the PIN domain, another important structural element of MCPIP1 is the C3H zinc finger domain, which is responsible for RNA binding. MCPIP1 recognizes stem loop sequences at the 3’ end of mRNAs and in pre-microRNAs (pre-miRNAs), and upon MCPIP1 binding, these molecules are degraded [17, 18].

Conditional knockout of MCPIP1 in keratinocytes impairs skin homeostasis in mice, which manifests phenotypically as decreased skin integrity in aged mice [19]. To initiate the development of tumors in Mcpip1^eKO^ mice, a single dose of the potent carcinogen 7,12-dimethylbenz[a]-anthracene (DMBA) was applied to the skin, followed by twice-weekly applications of the tumor-promoting agent 12-O-tetradecanoylphorbol-13-acetate (TPA) (following the protocol reported by Abel et al. [20]). After 12 weeks of DMBA/TPA treatment, both control and Mcpip1^eKO^ mice developed papillomas. However, this process was significantly accelerated in Mcpip1^eKO^ mice. Analysis of the mRNA profiles revealed elevated expression of typical markers for keratinocyte proliferation and tumor angiogenesis, which correlated with increased expression levels of IL-6, IL-33, and TGF-β. Furthermore, MCPIP1 RNase activity was observed to be essential for the negative regulation of genes encoding SCC antigens and matrix metallopeptidase 9 [8].

MiRNAs are very important regulatory factors in the etiology of skin cancers. They promote carcinogenesis through their direct involvement in the regulation of proliferation, invasion, angiogenesis, and apoptosis. Characterizing these molecules is an important part of molecular oncology research. The identification of miRNAs in tumors is important not only for understanding their function in modulating gene expression during carcinogenesis but also because of their potential utility as important markers for identifying tumors and predicting the course of the disease [21].

Since both mRNAs and pre-miRNAs are the targets of MCPIP1, in this work, we undertook a comprehensive analysis of the miRNA‒mRNA regulatory network in the developing papillomas of Mcpip1^eKO^ mice. This approach allowed us to understand the global course of changes at the transcriptome level and identify key genes and molecular pathways involved in the development of cSCC in humans.

## Materials and methods

### Mice

Mice with keratinocyte-specific Mcpip1 knockout (Krt14^Cre^Mcpip1^loxP/loxP^, herein referred to as Mcpip1^eKO^) were used in this study, as we previously described [8, 19, 22, 23]. All mice were on a C57BL/6 background. The regimen for DMBA-induced and TPA-promoted skin carcinogenesis was performed as we previously described [8]. In brief, 6- to 8-week-old mice were shaved and treated the following day with a single dose of 30 µg DMBA (Sigma‒Aldrich; Saint Louis, MO, USA). Two weeks after DMBA application, 15 µg of TPA (Sigma‒Aldrich) was applied topically twice per week for the next 10 weeks. The papillomas of Mcpip1^eKO^ and control mice were collected, immediately frozen in liquid nitrogen, and stored at -80 °C prior to RNA isolation.

### RNA sequencing (RNA-Seq)

Total RNA was isolated from papillomas of control and Mcpip1^eKO^ mice *via* homogenization in TRI Reagent (Sigma‒Aldrich). RNA samples were subjected to DNase I treatment and concentration using RNA Clean and Concentrator-5 (Zymo Research, Irvine, CA, USA). Quality control of RNA samples and RNA-Seq were performed by CeGaT GmbH (Tübingen, Germany). All samples passed quality control testing with a Qubit fluorometer (Thermo Fisher Scientific, Waltham, MA, USA) and a Bioanalyzer system (Agilent, Santa Clara, CA, USA). Next, 100 ng of total RNA was used for library preparation for long RNA-Seq, and 1000 ng was used for small RNA-Seq. The libraries were prepared using a KAPA RNA HyperPrep Kit with RiboErase (HMR) Globin (Roche, Basel, Switzerland) Kit for long RNA-Seq and with a NEXTFlex Small RNA-Seq Kit v3 (Bio Scientific, London, United Kingdom) for small RNA-seq. RNA-Seq was performed on a NovaSeq 6000 instrument with four independent biological replicates. For long RNA-seq, paired-end 100 bp reads were obtained, and for small RNA-seq, single-end 50 bp reads were generated. Demultiplexing of the sequencing reads was performed with Illumina bcl2fastq (2.20). Adapters were trimmed with Skewer (version 0.2.2) [24].

### Bioinformatic analysis of RNA-Seq data

The quality of the sequencing data (raw and trimmed fastq files) was validated using the FastQC (v.0.11.9) tool [25]. Quality reports were generated in multiqc (v. 1.12) [26]. Trimmomatic (v.0.39) [3] was used for long RNA sequence read trimming (parameters TruSeq3-PE-2.fa:2:30:10:8, SLIDINGWINDOW:4:15). Genome data (genome and annotation: *Mus musculus* GRCm39.106) were downloaded from Ensembl (accessed 23.06.2022). For long RNA-Seq analysis, the *Mus musculus* genome was indexed with STAR (v. 2.7.10a) [27]. STAR was also used to map clean reads to the reference genome. Long RNA read counting was performed in R (v.4.1.2) using the featureCounts function in the Rsubread package (v.2.8.2) [28] and gff annotation for genes and ncRNA_genes. For small RNA-Seq analysis, the genome reads were mapped to the reference genome using Bowtie2 (v.2.2.5) [29] with the following parameters: -N 0-L 18. Sambamba (v.0.6.6) [30] and BamTools (v.2.5.1) [31] were used to convert sam files to bam files and generate statistics from bam files, respectively. miRNA read counting was performed in R using featureCounts [28] with the following annotations: mirgeneDB 2.1 (https://mirgenedb.org/download; accessed: 8.07.2022) and miRBase Release 22.1 (https://www.mirbase.org/ftp.shtml; accessed: 8.07.2022).

Differential expression analysis of long RNAs and small RNAs was performed in R using DESeq2 (v.1.34.0) [32]. We next retrieved protein-coding genes (PCGs) from the list of differentially expressed long RNAs (DE-long RNAs) for further analyses. To retrieve the gene symbols of the putative orthologous PCGs between *Mus musculus* and *Homo sapiens*, the BioMart tool (R package biomaRt version 2.58) provided by Ensembl was used [33]. Functional Gene Ontology and KEGG pathways enrichment analyses were performed using the R package ClusterProfiler version 4.4 [34]. Volcano plots and dot plots were created using the ggplot2 libraries in R. Venn diagrams were generated using a freely available online tool (http://bioinformatics.psb.ugent.be/webtools/Venn). Gene expression data are presented in heatmaps as z scores, which were computed for each gene by subtracting the mean of the normalized RPKM value from individual RPKM value and then dividing by the standard deviation.

### Prediction of mRNA targets of miRNAs

To computationally predict miRNA interactions with PCGs, the DIANA-microT-CDS algorithm version 5.0 was used [35]. The DIANA-microT-CDS algorithm was executed utilizing annotations from Ensembl and miRBase. The microT score threshold was set to 0.7 for both human and mouse species. The targets of the miRNAs were also combined with the catalog of experimentally supported interactions included in the DIANA-TarBase v7.0 database [36]. Analyses of miRNA secondary structures were performed using the Vienna RNA web server [37].

### Quantitative reverse transcription polymerase chain reaction (RT‒qPCR)

RNA was isolated from human keratinocytes using TRI Reagent (Sigma‒Aldrich) and from BCC samples and mouse papillomas *via* homogenization in Fenozol (A&A Biotechnology, Gdansk, Poland). The total concentration and purity of RNA were determined by measuring the absorbance using a NanoDrop 2000 spectrophotometer (Thermo Fisher Scientific).

The expression levels of mRNAs were measured by RT-qPCR as we described previously [19]. Real-time quantitative PCR was performed with gene-specific primers using SYBR Green Master Mix (A&ABiotechnology). The sequences of the primers (Sigma-Aldrich) are listed in Table S1 (Additional file 1).

The expression levels of miRNAs were measured using a miRCURY LNA miRNA SYBR Green PCR System (QIAGEN) according to the manufacturer’s protocol. In brief, 100 ng of RNA was reverse transcribed using a miRCURY LNA RT Kit (QIAGEN). The synthesized cDNA was diluted eightfold prior to RT‒qPCR analysis. The expression levels of miRNAs were normalized to the miR-103a-3p expression level and calculated using the comparative Ct method and are presented as 2^− dCt^ values or as fold changes. miR-103a-3p was selected as a normalization control, as it was previously reported to be constitutively expressed in several normal and tumor tissues [38, 39]. Melting curves were generated at the end of each the RT‒qPCR analysis to verify the quality of the amplification products. The sequences of the primers used for amplification of the following miRNAs are available at www.qiagen.com: miR-103a-3p (no. YP00204063), miR-223-3p (no. YP00205986), miR-139-5p (no. YP00205874), miR-376c-3p (no. YP00204442), miR-7-5p (no. YP02119317), miR-92b-3p (no. YP00204384), and miR-196a-5p (no. YP00204386). Analyses of miRNA expression were performed with a QuantStudio3 thermocycler (Thermo Fisher Scientific).

### Cell culture

HaCaT human epidermal keratinocytes [40] and HEK293 cells (ATCC; Manassas, VA, USA) were cultured in Dulbecco’s modified Eagle’s medium (DMEM), A431 human epidermal carcinoma cells (ATCC) were cultured in low-glucose DMEM, and SCC-25 squamous carcinoma keratinocytes (ATCC) were cultured in DMEM: F12 supplemented with 400 ng/ml hydrocortisone. All media were supplemented with 10% fetal bovine serum. The cell cultures were maintained at 37 °C in a 5% CO_2_ humidified atmosphere. To stably knock down MCPIP1 in A431 cells, lentiviral vectors expressing a control shRNA or a shRNA specific for MCPIP1 were used (Sigma‒Aldrich), as described previously [8].

### Transfection with miRNA mimics

SCC-25 cells were seeded in 6-well plates at 8 × 10^5^ cells per well 24 h prior to transfection with one of the following miRNA mimics (10 nM; QIAGEN, Germantown, MD, USA): negative control (NC) mimic (YM00479902), hsa-miR-376c-3p mimic (YM00471227), hsa-miR-223-3p mimic (YM00471011) or hsa-miR-139-5p mimic (YM00471080). Mimic transfection was performed using jetPRIME reagent (Polypus, London, UK). Seventy-two hours after transfection, cells were collected for western blot and flow cytometric analyses of cell cycle profiles.

### Transfection with pre-miRNA expressing vectors

Sequences encoding human pre-miRNA-223, -376c and − 139 were cloned into pcDNA3.0 vector. For that, complementary oligonucleotides were synthesized (Sigma-Aldrich; Table S1 (Additional file 1)), denatured, annealed and ligated into pcDNA3.0 plasmid at BamHI and EcoRI sites. HEK293 cells were seeded in 12-well plates at 10^5^ cells per well 24 h prior co-transfection with 400 ng of pcDNA3.0 pre-miRNA plasmid and 400 ng of pcDNA3.0 empty, pcDNA3.0-MCPIP1, or pcDNA3.0-MCPIP1-D141N plasmid using jetPRIME Reagent. Twenty four hours after transfection, cells were harvested in TRI (for RNA isolation and RT-qPCR analysis) or RIPA buffer (for western blot analysis).

### Expression and immunoprecipitation of 3xFLAG MCPIP1

The doxycycline-dependent TetON system was used (pLIX vectors) to obtain HaCaT cells overexpressing 3xFLAG-MCPIP1-WT, 3xFLAG-MCPIP1-D141N, or empty control. To induce expression of exogenous protein, 1 µg/ml of doxycycline (BioShop, Burlington, Canada) was added to the medium 24 h after seeding cells. For immunoprecipitation, cells grown at sub-confluency were harvested by trypsinization 24 h after treatment with doxycycline and lysed in IP lysis buffer (0,5% NP-40, 150 mM NaCl, 50 mM Tris at pH 7.5 with protease and phosphatase inhibitors). IP was performed with anti-FLAG M2 (#F1804, Sigma-Aldrich) coated Protein G Dynabeads (Invitrogen, Waltham, MA, USA) for 1 h at 4^0^C rolling. Following washes, the beads with purified proteins were utilized for degradation assays. Alternatively, the beads were mixed with 2X SDS sample buffer and boiled for 5 min at 65 ^0^C to elute proteins for western blot analysis.

### Degradation assays

Transcript fragments containing pre-miRNA sequences were synthesized with T7 RNA polymerase kit (TranscriptAid T7 high Yield Transcription Kit; Thermo Fisher Scientific) using linearized pcDNA3.0-pre-miRNA vector as a template. Synthesized RNAs were purified using RNA Clean and Concentrator-5 (Zymo Research, Irvine, CA, USA) and stored at -80 ^0^C. RNA cleavage assays were performed with immunoprecipitated 3xFLAG-MCPIP1-WT or -DN and 500 ng of in vitro transcribed pre-miRNAs at 37 °C in cleavage buffer (50 mM Tris, 150 mM NaCl, 2.5 mM MgCl_2_, 2.5 mM DTT, 0.5 mM EDTA, 0.025 mM ZnCl_2_ at pH 8.3). Reaction products were separated on 8% denaturing polyacrylamide gel electrophoresis in TBE buffer. Subsequently, the gel was stained with SimplySafe (EurX, Gdansk, Poland) and visualized using a ChemiDoc MP imaging system (BioRad).

### Western blot analysis

Cells were lysed in RIPA buffer supplemented with Complete Protease Inhibitor Cocktail (Roche) and PhosSTOP Phosphatase Inhibitor Cocktail (Roche). The protein concentration was measured with a bicinchoninic acid assay. Proteins in the cell lysates were separated by SDS–PAGE on polyacrylamide gels and transferred to PVDF membranes (Merck Millipore, Billerica, MA, USA). The membranes were blocked for 1 h in 5% nonfat milk in Tris-buffered saline with 0.05% Tween 20 (BioShop) and incubated with the appropriate primary antibody at 4 °C. For detection, Immobilon Western Chemiluminescent HRP Substrate (Merck Millipore) and a ChemiDoc imaging system (Bio-Rad, Hercules, CA, USA) were used. The cell Cycle Regulation Antibody Sampler Kit (#9932) and Cell Cycle Regulation Antibody Sampler Kit II (#9870) were obtained from Cell Signaling Technology (Beverly, MA, USA), and anti-FLAG M2 (Sigma-Aldrich). For detecting human MCPIP1 protein, the in-house antibody was used as previously described [8]. Densitometric quantification was performed using ImageLab (Bio-Rad) with β-actin (Sigma‒Aldrich, no. A1978) as an endogenous control. Relative protein expression values are presented as fold changes.

### Flow cytometry

Seventy-two hours after transfection with miRNA mimics, cells were harvested and fixed in ice-cold 70% ethanol. The samples were stored at 4 °C prior to staining with FxCycle™ PI/RNase Staining Solution (Thermo Fisher Scientific) and analysis with an Attune NxT flow cytometer (Thermo Fisher Scientific).

### Patient tissue samples

For the analysis of miRNA profiles in BCC, skin biopsies obtained from 12 patients with clinically diagnosed and histologically confirmed BCC were used as material for the study. All patients (4 women and 8 men, age range: 61–98 years, mean: 73.17 ± 9.57 years) were surgically treated at the Department of Dermatology, Venereology and Allergology of Wroclaw Medical University in Wroclaw, Poland. All biopsies were obtained under local anesthesia with 2% lidocaine with adrenaline, and most were from lesions on sun-exposed areas of the face and neck (nose, cheeks, forehead, and neck). Only 3 biopsies were obtained from lesions on the trunk. Skin samples from healthy controls were collected from patients who underwent a surgical procedure for non-malignant skin lesions localized on the trunk.

### Immunohistochemistry staining of cSCC specimens

Skin biopsies from cSCC patients and IHC staining were performed as described previously [8]. The following antibodies were used: mouse CD3 (Thermo Fisher Scientific, no. 14-0037-82), mouse PCNA (Dako, Hamburg, Germany, no. M0879), mouse CD68 (Abcam, Cambridge, UK, no. ab955). Sections were examined using a Leica DMC5400 Microscope (Leica Microsystems, Wetzlar, Germany) and figures were prepared using ImageJ (National Institutes of Health, Bethesda, MD).

### Statistics

For RT‒qPCR, western blot and flow cytometry data, the groups were compared by one-way analysis of variance (ANOVA) or unpaired Student’s t test using GraphPad Prism 10 (GraphPad, La Jolla, CA, USA), with *P* < 0.05 considered to indicate statistical significance. All numeric data are presented as the mean ± standard error of the mean (SEM) values. The figures were created using CorelDRAW 2023 (Corel Corporation, ON, Canada).

## Results

### Loss of keratinocyte MCPIP1 elicits profound changes in RNA expression profiles in chemically induced skin papillomas

**Fig. 1:**
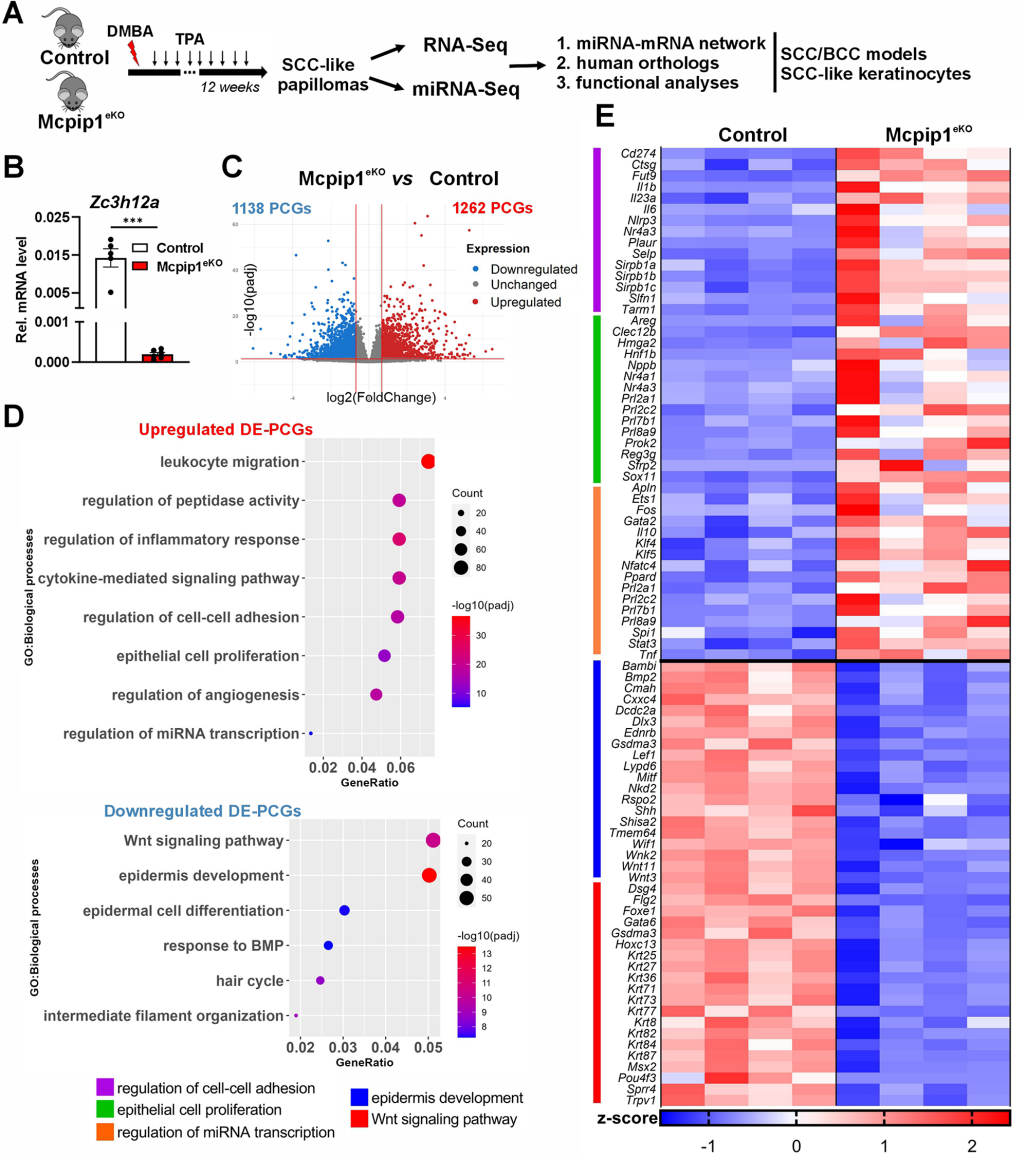
Whole-transcriptome profiling of Mcpip1^eKO^papillomas. (**A**) A schematic showing the DMBA/TPA protocol used to induce papilloma formation and an outline of the RNA-Seq and follow-up analyses pipeline. (**B**) RT‒qPCR analysis of *Zc3h12a* mRNA in control and Mcpip1^eKO^ papillomas (*n* = 5–6). *EF2* was used as an internal control. *** – *P* < 0.001 by Student’s t test. (**C**) Volcano plot showing the DE-PCGs between control and Mcpip1^eKO^ papillomas. Adjusted *P* value (padj) < 0.05, fold change (FC) > 1.6. (**D**) GO enrichment analysis of upregulated biological processes enriched in the upregulated and downregulated DE-PCGs. (**E**) Heatmap showing the expression levels (z scores) of representative DE-PCGs related to the selected GO terms. The top 15 DE-PCGs (based on FC scores) in each GO term are shown. DE, differentially expressed; DMBA, 7,12-dimethylbenz[a]-anthracene; GO, Gene Ontology; PCG, protein-coding gene; SCC, squamous cell carcinoma; TPA, 2-O-tetradecanoylphorbol-13-acetate

Total RNA was isolated from chemically induced papillomas of four control and four Mcpip1^eKO^ mice. Subsequently, RNA-Seq was performed to identify differentially expressed protein-coding genes (DE-PCGs) and differentially expressed miRNAs (DE-miRNAs) from the same RNA samples, with a FC of > 1.6 and an adjusted (adj.) *P* value of < 0.05 as the cutoff values (Fig. 1A). As expected, the level of *Zc3h12a* mRNA was almost completely decreased in the papillomas of the keratinocyte-specific Mcpip1 knockout mice compared to the control ones (Fig. 1B). RNA-Seq analysis revealed widespread transcriptome changes in Mcpip1^eKO^ papillomas, with a total of 2400 DE-PCGs identified (Fig. 1C). Overall, the deep RNA-Seq technique used in the current study enabled us to identify ~ 3 times more DE-PCGs than were identified in a previous expression analysis with the Ion AmpliSeq panel [8]. Gene Ontology enrichment analyses were subsequently performed to identify the biological processes influenced by the upregulated and downregulated DE-PCGs. As we previously showed [8], processes related to the inflammatory response, cytokine-mediated signaling pathway, regulation of peptidase activity, and angiogenesis were significantly enriched with the upregulated DE-PCGs in Mcpip1^eKO^ papillomas. In addition, the present analysis revealed that groups of upregulated DE-PCGs were functionally annotated to the following biological processes: “regulation of cell‒cell adhesion” (i.e., *Cd274*,* Ctsg*,* Il6*,* Nlrp3*,* Selp*, and *Sirpb1a/b/c*), “epithelial cell proliferation” (i.e., *Areg*,* Hmga2*,* Nppb*,* Nr4a1/3*,* Sfrp2*, and *Sox11*), and “regulation of miRNA transcription” (i.e., *Ets1*,* Fos*,* Gata2*,* Klf4/5*,* Ppard*, and *Spi1*) (Fig. 1D-E and Fig. S1A, Additional file 2). We previously found that 261 were downregulated in Mcpip1^eKO^ papillomas but no functional enrichment of these genes was identified. The current study provides a better understanding of the functional relevance of the downregulated DE-PCGs in Mcpip1^eKO^ papillomas. In particular, we identified genes associated with the following biological processes: “Wnt signaling pathway” (i.e., *Bambi*,* Bmp2*,* Dlx3*,* Mitf*,* Shh*, and *Wnt3/11*), “epidermis development” (*Dsg4*,* Flg2*,* Krt25/36/71/73*,* Sprr4*, and *Trpv1*), “response to BMP” (i.e., *Bmp2/4/6*,* Cer1*,* Dlx3*,* Lef1*,* Msx1/2*, and *Smad6*), “hair cycle” (i.e., *Edar*,* Ngfr*,* Foxe1*,* Trpv1*, and *Shh*), and “intermediate filament organization” (i.e., *Krt31/33b/36/39/71/84/87*) (Fig. 1D-E and Fig. S1B, Additional file 2).

### Loss of MCPIP1 affects miRNA expression profiles in skin malignancies

**Fig. 2:**
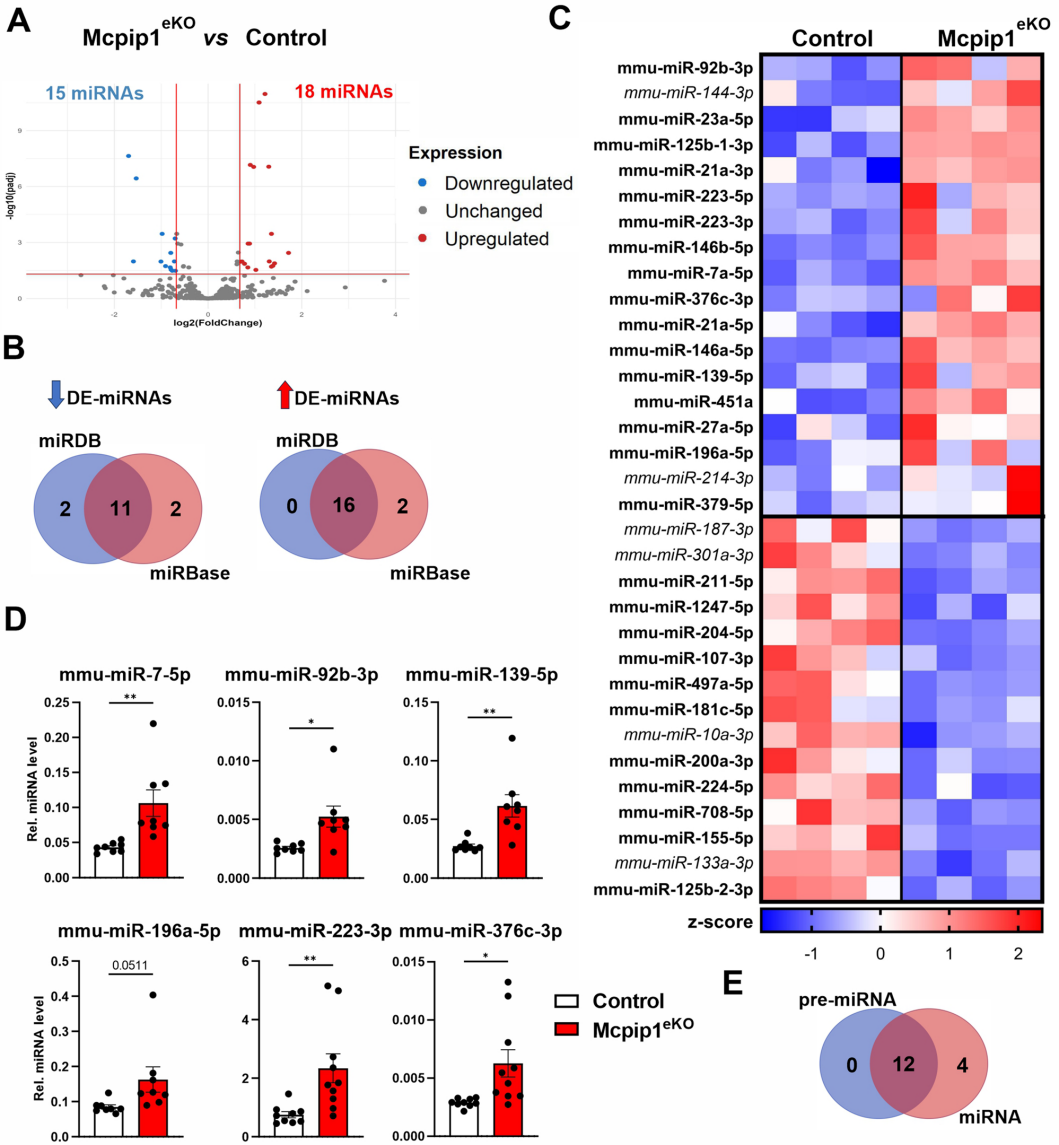
Global characterization of the miRNA expression profile in Mcpip1^eKO^papillomas. (**A**) Volcano plot showing the DE-miRNAs between control and Mcpip1^eKO^ papillomas. Adjusted *P* value (padj) < 0.05, fold change (FC) > 1.6. (**B**) Comparison of the total number of downregulated and upregulated in Mcpip1^eKO^ DE-miRNAs annotated in miRDB (blue) and miRBase (red). (**C**) Heatmap showing the expression levels (z scores) of all the DE-miRNAs in Mcpip1^eKO^ papillomas. MiRNAs annotated in both miRBase and miRDB are presented in bold font. MiRNAs annotated in only one database are presented in italic font. (**D**) RT‒qPCR analysis of mmu-miR-7-5p, mmu-miR-92b, mmu-miR-139-5p, mmu-miR-196a-5p, mmu-miR-223-3p, and mmu-miR-376c-3p expression (*n* = 8). MiR-103a-3p was used as an internal control. * – *P* < 0.05; ** – *P* < 0.01 by Student’s t test. (**E**) Comparison of the total number of upregulated in Mcpip1^eKO^ DE-miRNAs (red) and DE-pre-miRNAs (blue). DE, differentially expressed; Mmu, *Mus musculus*

Analysis of the global differential expression of miRNAs integrated with miRNA annotations obtained from the miRBase and miRGene databases (miRDB) revealed 15 significantly downregulated and 18 upregulated miRNAs in Mcpip1^eKO^ papillomas compared to the control, with a FC of > 1.6 and an adj. *P* value of < 0.05 as the cutoff values (Fig. 2A-C). To select only robustly annotated miRNAs, only miRNAs that were annotated in both miRBase and miRGene (16 upregulated and 11 downregulated miRNAs) were considered in the follow-up validation experiments.

The 16 miRNAs that were significantly upregulated in chemically induced skin papillomas of Mcpip1^eKO^ mice included miR-7, miR-21, and miR-146a, whose biogenesis was previously reported to be negatively regulated by the MCPIP1 RNase [18] (Fig. 2C). To validate the results of DE miRNAs analysis, RT‒qPCR was subsequently performed on a larger number of samples (*n* = 8). The differential expression of 5 selected miRNAs, i.e., miR-92b-3p, miR-139-5p, miR-196a-5p, miR-223-3p, and miR-376c-3p, and the mouse ortholog of human miR-7 (as a control) between control and Mcpip1^eKO^ papillomas was validated (Fig. 2D).

We speculated that the most plausible mechanism of their upregulation would involve a direct nucleolytic cleavage mediated by MCPIP1 RNase of the corresponding pre-miRNAs, as the levels of their precursor RNAs were significantly increased in the Mcpip1^eKO^ papillomas. Overall, 75% of the 16 shortlisted upregulated miRNAs were also upregulated on the level of the pre-miRNA (Fig. 2E and Table S2, Additional file 3).

### Downregulation of MCPIP1 increases the expression of miR-223-3p, miR-376c-3p and mir-139-5p in human keratinocyte carcinomas

**Fig. 3:**
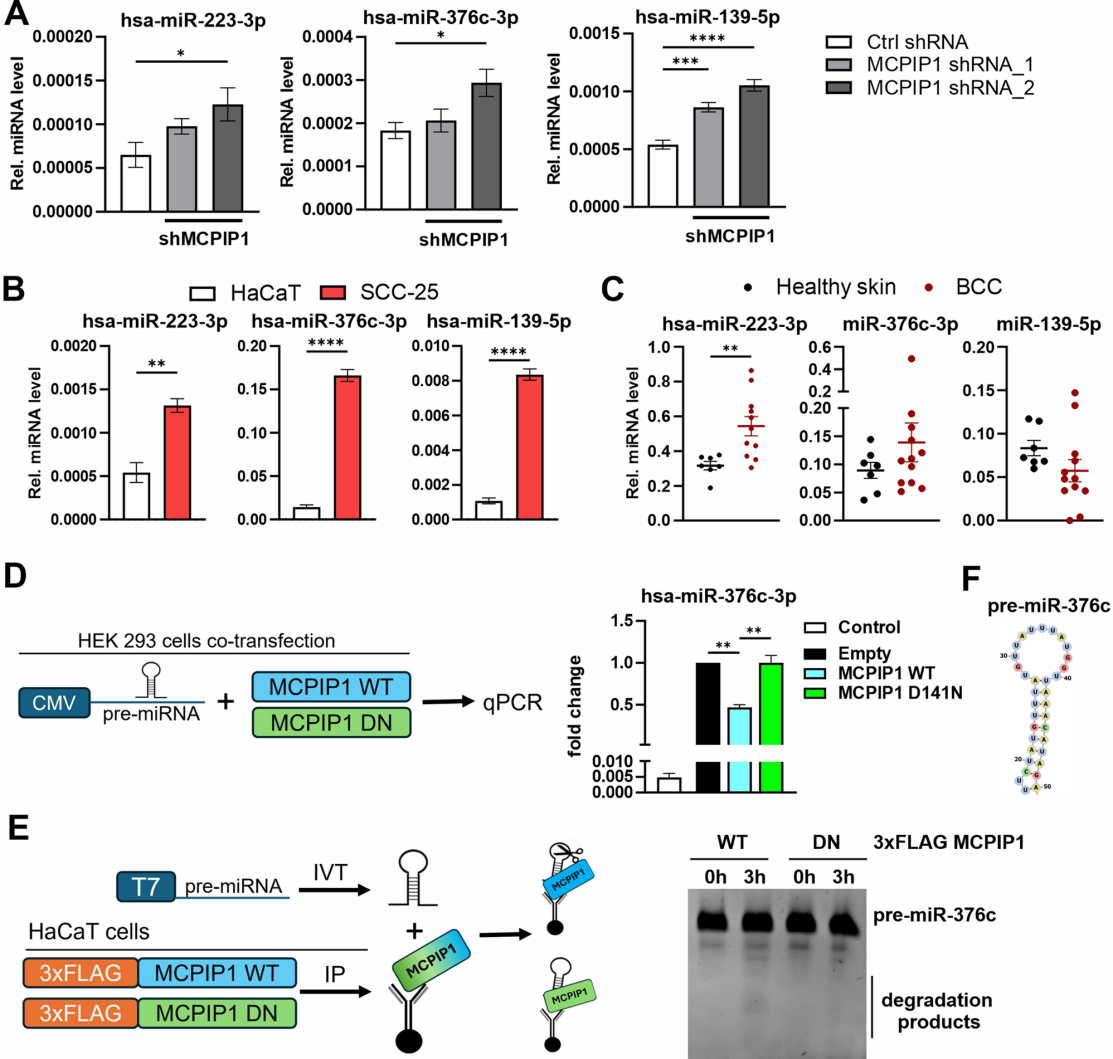
Expression of miR-223-3p, miR-376c-3p, and miR-139-5p in human keratinocyte carcinomas and regulation of their biogenesis by MCPIP1. (**A**) RT‒qPCR analysis of hsa-miR-223-3p, hsa-miR-376c-3p and hsa-miR-139-5p expression in A431 cells expressing the control (NC, negative control) or MCPIP1-specific shRNA (*n* = 5). (**B**) RT‒qPCR analysis of miRNA expression in HaCaT and SCC-25 keratinocytes (*n* = 4). (**C**) RT‒qPCR analysis of miRNA expression in skin sections from BCC patients (*n* = 12) and healthy individuals (*n* = 7). (**D**) HEK293 cells were co-transfected with pcDNA3.0 (control), pcDNA3.0 MCPIP1 or pcDNA3.0 MCPIP1-D141N and pcDNA3.0 expressing pre-miR-376c. **Left panel**: Scheme indicating experimental design. **Right panel**: RT-qPCR analysis of miRNA expression (*n* = 3). (**E**) Degradation assays were performed using 3xFLAG MCPIP1 or 3xFLAG MCPIP1-D141N immunoprecipitated from HaCaT cells with in vitro transcribed pre-miR-376c as a substrate. **Left panel**: Scheme indicating experimental design. **Right panel**: Image of RNA electrophoresis in 8% PAA gel. (**F**) Predicted secondary structure of hsa-pre-miR-376c. MiR-103a-3p was used as an internal control. * – *P* < 0.05; ** – *P* < 0.01, *** – *P* < 0.001, **** – *P* < 0.0001 by Student’s t test (**B**, **C**) or one-way ANOVA (**A**, **D**). Hsa, *Homo sapiens*

To determine whether MCPIP1 regulates the biogenesis of the 5 pre-selected miRNAs in human cells, the expression levels of these miRNAs were subsequently analyzed *via* RT‒qPCR. As a model, we used A431 human epidermal carcinoma cells stably expressing shRNA against MCPIP1, which we described previously [8]. We found that in these malignant human keratinocytes, the relative expression of miR-223-3p, miR-376c-3p, and miR-139-5p but not that of miR-92b or miR-196a-5p was inversely correlated with the MCPIP1 protein level (Fig. 3A and Fig. S2A-B, Additional file 4). Thus, in the context of skin carcinogenesis, miR-223-3p, miR-376c-3p and miR-139-5p were identified as novel miRNAs whose expression is modulated by MCPIP1 activity in both murine and human models.

The levels of miR-223-3p, miR-376c-3p, and miR-139-5p were next measured in human keratinocyte carcinoma cells and tissues. The expression levels of all the investigated miRNAs were significantly (*P* value < 0.05) higher in SCC-25 malignant SCC keratinocytes than in noncancerous HaCaT keratinocytes (Fig. 3B). Moreover, for BCC, our RT‒qPCR analysis of normal skin and carcinoma tissues indicated significantly increased expression of miR-223-3p but not of miR-376c-3p nor miR-139-5p (Fig. 3C).

To obtain mechanistic understanding of the role of MCPIP1 in the regulation of miR-223-3p, miR-376c-3p, and miR-139-5p, we cloned corresponding pre-miRNAs into expression vector. Subsequently, we found that overexpression of the wild type MCPIP1 (WT), but not of the catalytically inactive MCPIP1-D141N mutant (D141N), downregulated the level of mature miR-376c-3p in cells ectopically expressing pre-miR-376c sequence. This was not observed in case of miR-223-3p nor miR-139-5p (Fig. 3D and Fig. S2C-D, Additional file 4). Moreover, an in vitro degradation assay with 3xFLAG-MCPIP1 RNase and an RNA fragment containing pre-miR-376c motif as a substrate revealed presence of degradation products after 3 h incubation with WT but not with the D141N mutant (Fig. 3E and Fig. S2E-F, Additional file 4). Thus, we conclude that MCPIP1 modulates the biogenesis of miR-376c-3p *via* direct nucleolytic cleavage withing the loop region of the corresponding pre-miRNA (Fig. 3F), whereas in case of miR-223-3p and miR-139-5p the regulation is most likely indirect.

### Overexpression of miR-223-3p, miR-376c-3p and mir-139-5p advances the cell cycle in malignant human keratinocytes

**Fig. 4:**
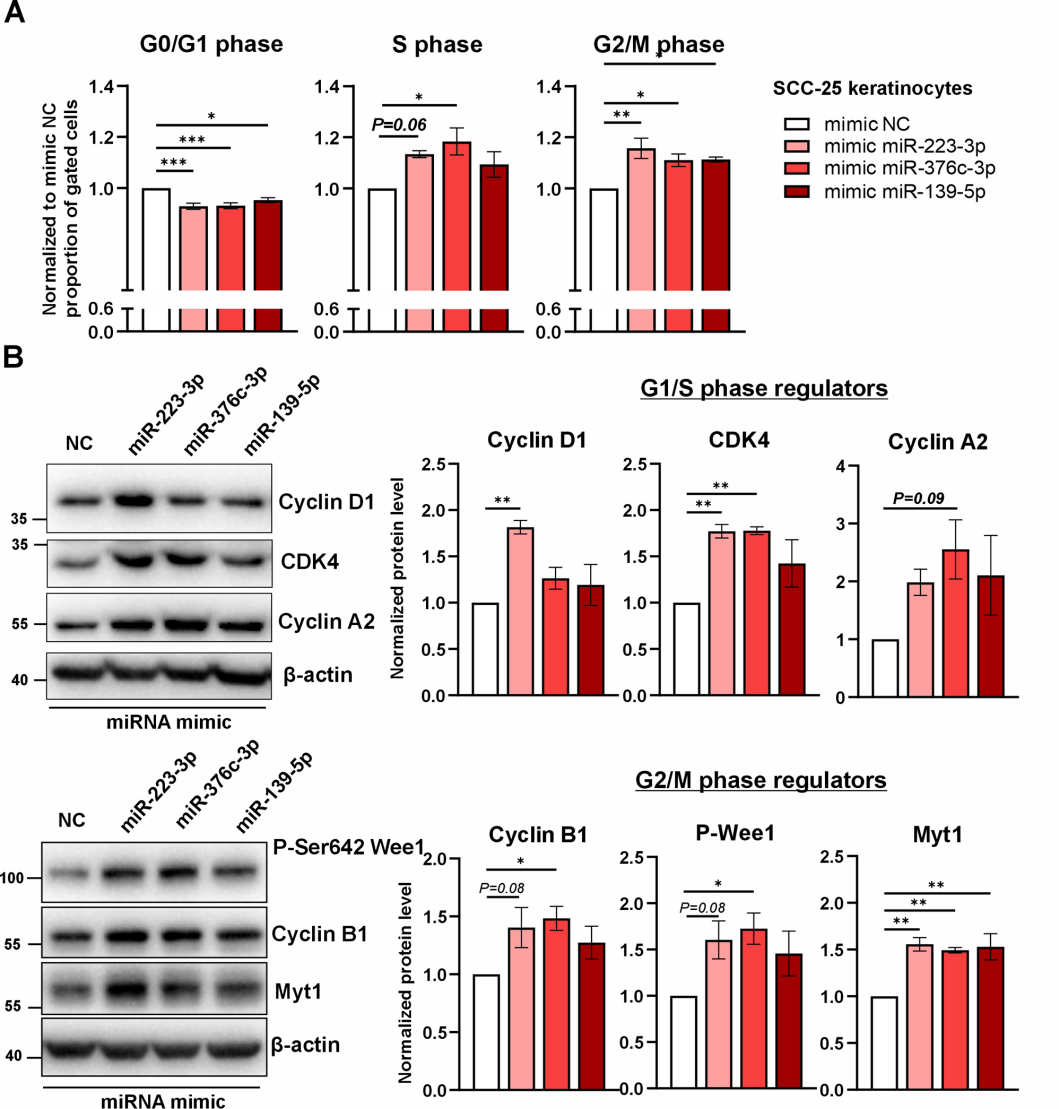
Overexpression of miR-223-3p, miR-376c-3p, and miR-139-5p mimics in SCC-25 keratinocytes. Cells were transfected with the appropriate miRNA mimics or NC mimics and analyzed 72 h later. (**A**) The graph shows the cell cycle distribution (G0/G1, S, and G2/M phases) based on propidium iodide staining (*n* = 4). (**B**) Western blot analysis and densitometric quantification of Cyclin D1, CDK4, Cyclin A2, P-Ser642 Wee1, Cyclin B1, and Myt1 expression. β-Actin was used as an internal loading control (*n* = 3–5). * – *P* < 0.05; ** – *P* < 0.01, *** – *P* < 0.001 by one-way ANOVA. NC, negative control

We next investigated whether increased expression of miR-223-3p, miR-376c-3p or miR-139-5p affects the proliferative activity of SCC-like keratinocytes. To this end, SCC-25 cells were transiently transfected with the appropriate miRNA mimics or NC mimics. Seventy-two hours after transfection, cell cycle profiles were analyzed using flow cytometric quantification of DNA staining with propidium iodide. Ectopic expression of all analyzed miRNAs significantly advanced the cell cycle in SCC cells, with the strongest effect observed for miR-223-3p and miR-376c-3p. Compared to that in the NC group, the proportions of G0/G1-phase cells in keratinocytes expressing the analyzed miRNA mimics were decreased. This decrease was accompanied by significant increases in the proportions of S-, G2-, and M-phase cells. In particular, compared to that in the NC group, the proportion of cells that progressed through the S/G2/M phases was increased by approximately 28% in the groups of SCC-25 cells expressing the miR-223-3p or miR-376c-3p mimic and by 19% in the group of cells expressing the miR-139-5p mimic (Fig. 4A).

The cell cycle profile variations between the groups, as determined on the basis of the population-based DNA content analysis, were subsequently confirmed by western blot analysis of key cell cycle regulators. The expression of cyclin D1, a critical cyclin that promotes cell proliferation, was significantly increased in SCC-25 cells expressing miR-223-3p but not in those expressing miR-376c-3p or miR-139-5p, suggesting that distinct molecular mechanisms are driven by these miRNAs in skin cancer. Interestingly, the expression of cyclin-dependent kinase 4 (CDK4), whose activation is critical for the transition from G1 to S phase and for the inhibition of senescence, was significantly increased by approximately 1.8-fold in miR-223-3p- or miR-376c-3p-overexpressing keratinocytes. Furthermore, the protein expression levels of key cyclins that cooperatively mediate the transition from S phase to G2 and M phases—cyclin A2 and cyclin B1—were elevated in SCC keratinocytes transfected with either the miR-223-3p or miR-376c-3p mimic. Finally, the activation of Wee1 and the expression of Myt1, which are critical mitotic checkpoint kinases, were increased upon transfection of the miRNA mimics. Overall, robust activation of cell cycle modulators was observed in SCC2-25 cells transfected with the miR-223-3p or miR-376c-3p mimic, whereas overexpression of miR-139-5p exerted a weaker effect (Fig. 4B). In addition, overexpression of miR-376c-3p mimic in SCC-25 led to increased transcriptional expression of genes encoding mesenchymal markers such as Vimentin, SNAIL, and of β-catenin (Fig. S3, Additional file 5).

### Bioinformatic analysis of the miRNA‒mRNA network in cutaneous malignancies driven by MCPIP1 deficiency

**Fig. 5:**
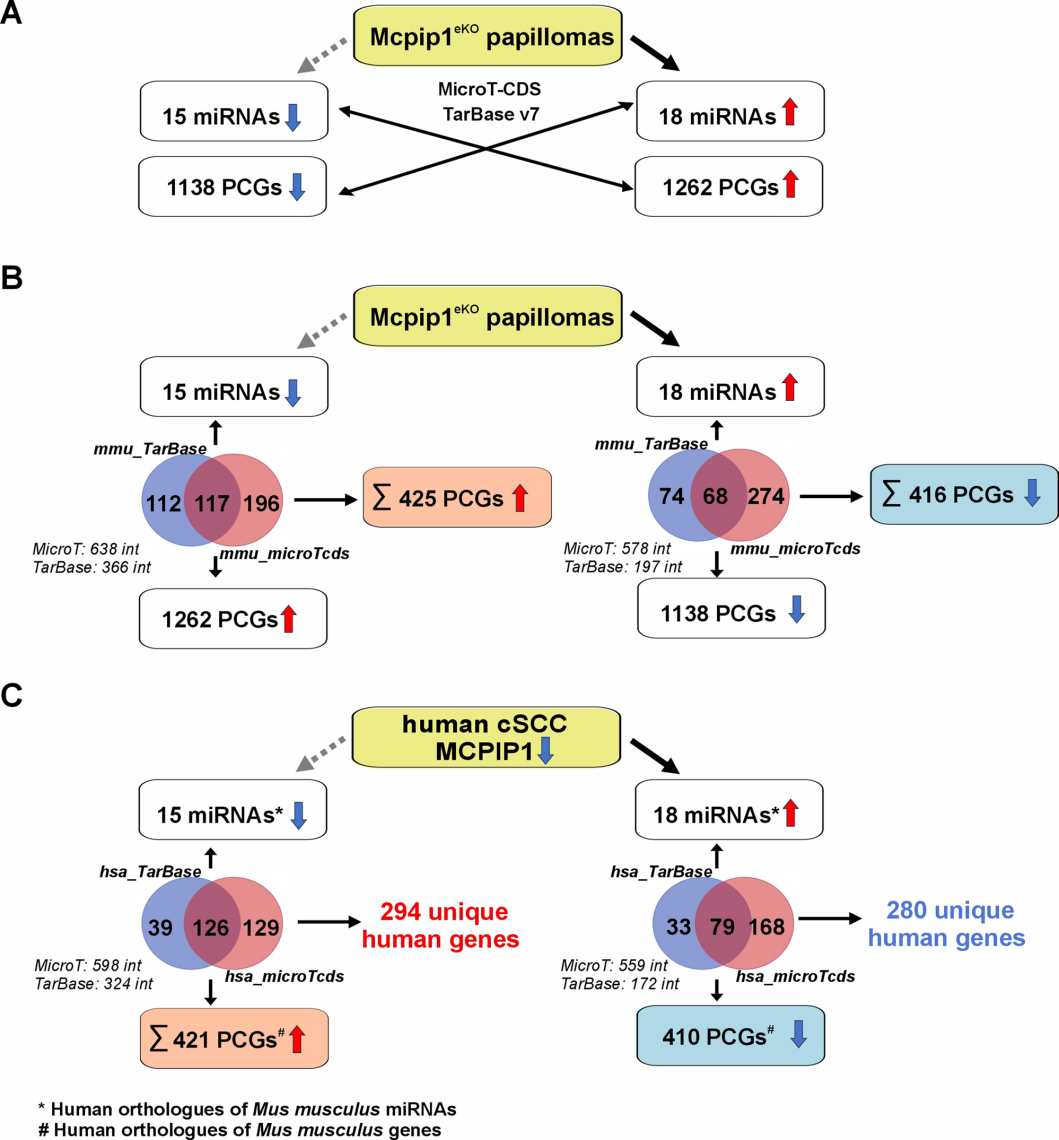
Identification of human orthologs of *Mus musculus *miRNA‒mRNA target pairs. (**A**) Schematic showing the pipeline for bioinformatic analysis of the miRNA‒mRNA network in Mcpip1^eKO^ papillomas. (**B**) MicroT-CDS and TarBase v7 predictions of miRNA target genes in Mcpip1^eKO^ papillomas. (**C**) Putative murine miRNA target gene symbols were converted into those of their human orthologs using the BioMart tool provided by Ensembl. The graph shows the numbers of putative miRNA target genes in humans. The dashed gray lines indicate indirect effects of MCPIP1 downregulation. The Venn diagrams show the unique genes identified in each comparison. The data in italics denote the total number of miRNA–mRNA interactions. Hsa, *Homo sapiens;* int, interaction; mmu, *Mus musculus;* PCG, protein-coding gene; SCC, cutaneous squamous cell carcinoma

We next sought to correlate the expression profiles of miRNAs and their putative target genes. The DIANA-microT-CDS algorithm was used to predict potential target genes of the DE-miRNAs. In addition, the DIANA-TarBase v7 database was used to determine the experimentally validated mRNA targets of the DE-miRNAs (Fig. 5A). Bioinformatic analyses were initially performed for murine genes that were identified *via* RNA-Seq as being differentially expressed between the Mcpip1^eKO^ and control groups, and these genes were subsequently matched to their human orthologs (Fig. 5B-C). For each comparison, the cumulative target interaction list containing both the predicted and validated miRNA target genes was generated. We identified 294 unique genes as putative targets of the 15 human orthologs of the downregulated miRNAs in the Mcpip1^eKO^ group and 280 genes as putative targets of the 18 upregulated miRNAs (Fig. 5C and Tables S3-S6, Additional files 6–9).

**Fig. 6:**
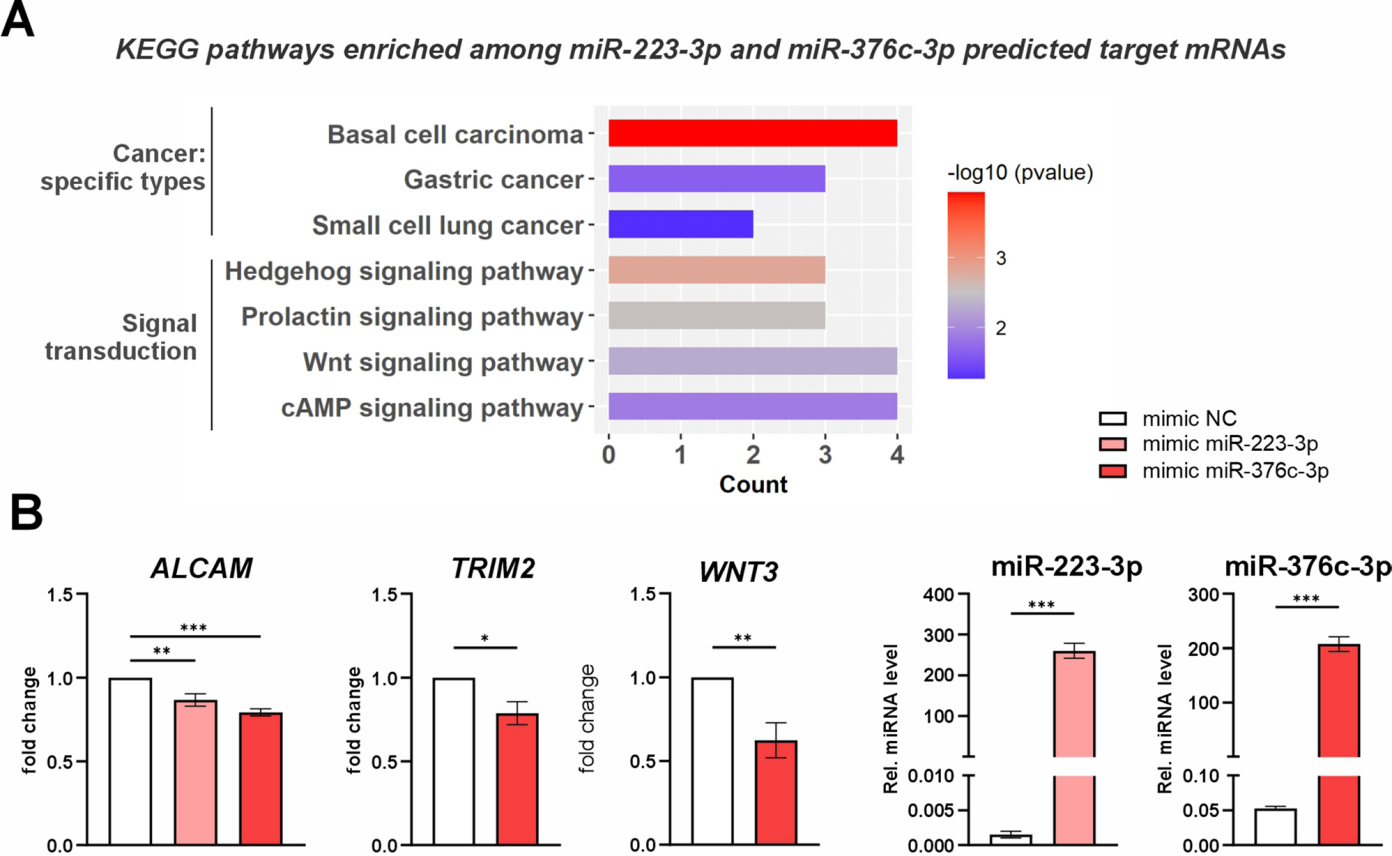
Network analysis of miR-223-3p and miR-376c-3p target mRNAs. (**A**) The results of KEGG pathway enrichment analysis of the putative miR-223-3p and miR-376c-3p target mRNAs. (**B**) RT-qPCR analysis of *ALCAM*,* TRIM2*, and *WNT3* mRNAs (*n* = 5) and of *miR-223-3p* and *miR-376c-3p* (*n* = 3) in SCC-25 cells transfected with 10nM of the control or miR-376c-3p mimic for 24 h. *EF2* or miR-103a-3p were used as an internal control. * – *P* < 0.05; ** – *P* < 0.01, *** – *P* < 0.001 by one way ANOVA or Student’s t test

Our functional analysis indicated that increased levels of miR-223-3p and miR-376c-3p specifically in SCC-like keratinocytes affect cell cycle progression, presumably contributing to cancer progression or invasion (Fig. 4). As a next step, we investigated the functional role of their putative mRNA interactors, selected on the basis of the bioinformatic predictions (Fig. 5). The KEGG pathway analyses of the 68 miR-223-3p and miR-376c-3p target genes indicated their connection with the specific types of cancer (including basal cell carcinoma) and with the signal transduction pathways, in particular of Hedgehog, Wnt and cAMP signaling (Fig. 6A). We next validated randomly selected miR-223-3p and miR-376c-3p predictions - *ALCAM*, *TRIM2*, and *WNT3*, by overexpression of miRNA mimics in SCC-25 cells followed by RT-qPCR (Fig. 6B).

**Fig. 7:**
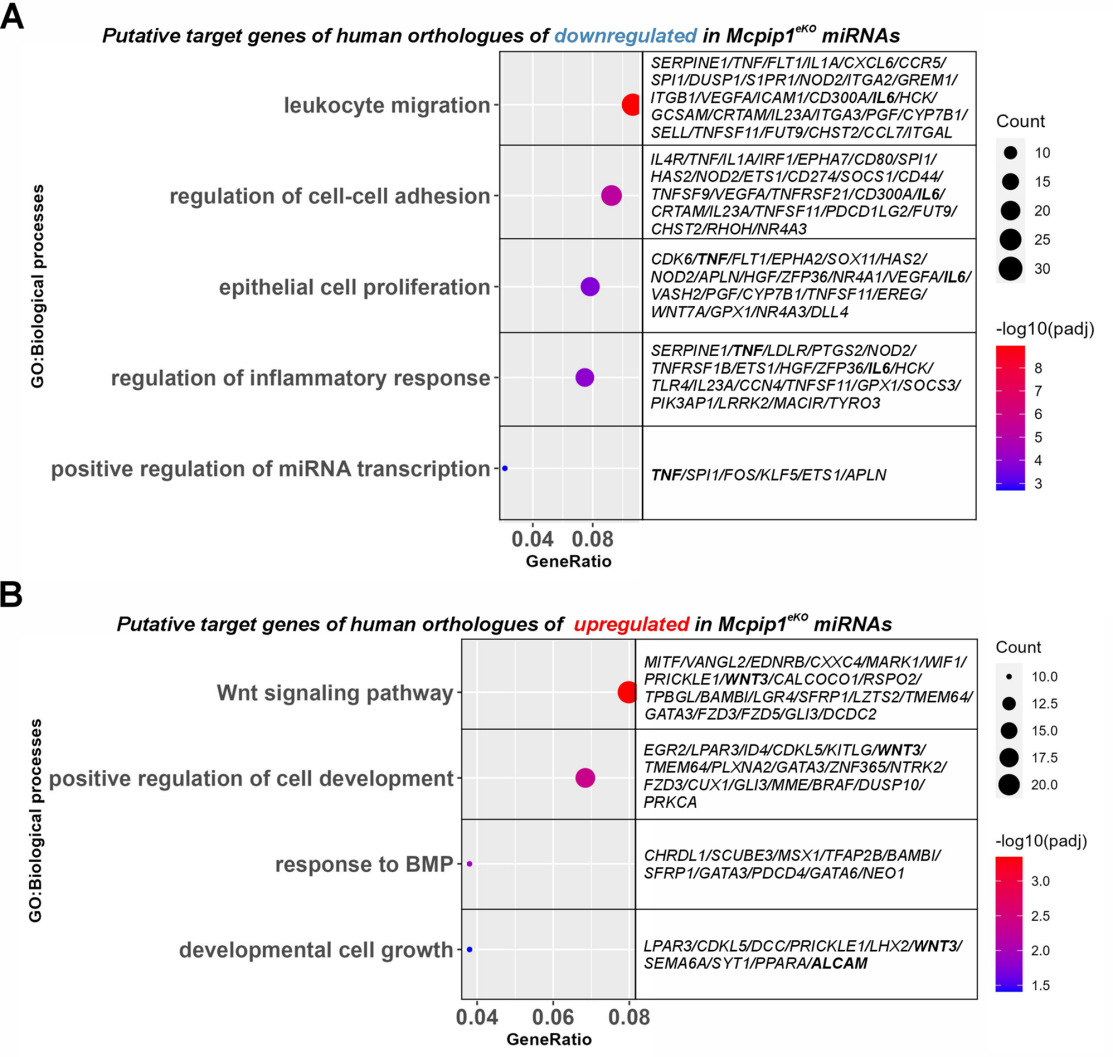
Functional enrichment analysis of the human miRNA target genes. The results of Gene Ontology (GO) analysis of the upregulated biological processes and a list of putative human target genes of the (**A**) downregulated in Mcpip1^eKO^ papillomas miRNAs and (**B**) upregulated in Mcpip1^eKO^ papillomas miRNAs. Selected GO terms are shown. GO, Gene Ontology

To obtain global insights into the functional role of the shortlisted miRNAs in humans, pathway enrichment analysis was performed on all the shortlisted 294 putative targets of the downregulated miRNAs and all the 280 putative targets of the upregulated miRNAs. To this end, the two clusters of genes were subjected to GO enrichment analysis. GO terms related to leukocyte migration, regulation of cell‒cell adhesion, epithelial proliferation, and regulation of the inflammatory response were the terms most enriched in the putative targets of the downregulated miRNAs. In addition, we identified a cluster of genes functionally annotated to the regulation of miRNA transcription (Fig. 7A). On the other hand, GO analysis indicated that the biological processes “Wnt signaling”, “regulation of cell development”, “response to BMP”, and “developmental cell growth” were the processes most enriched in the target genes of the upregulated miRNAs (Fig. 7B).

**Fig. 8:**
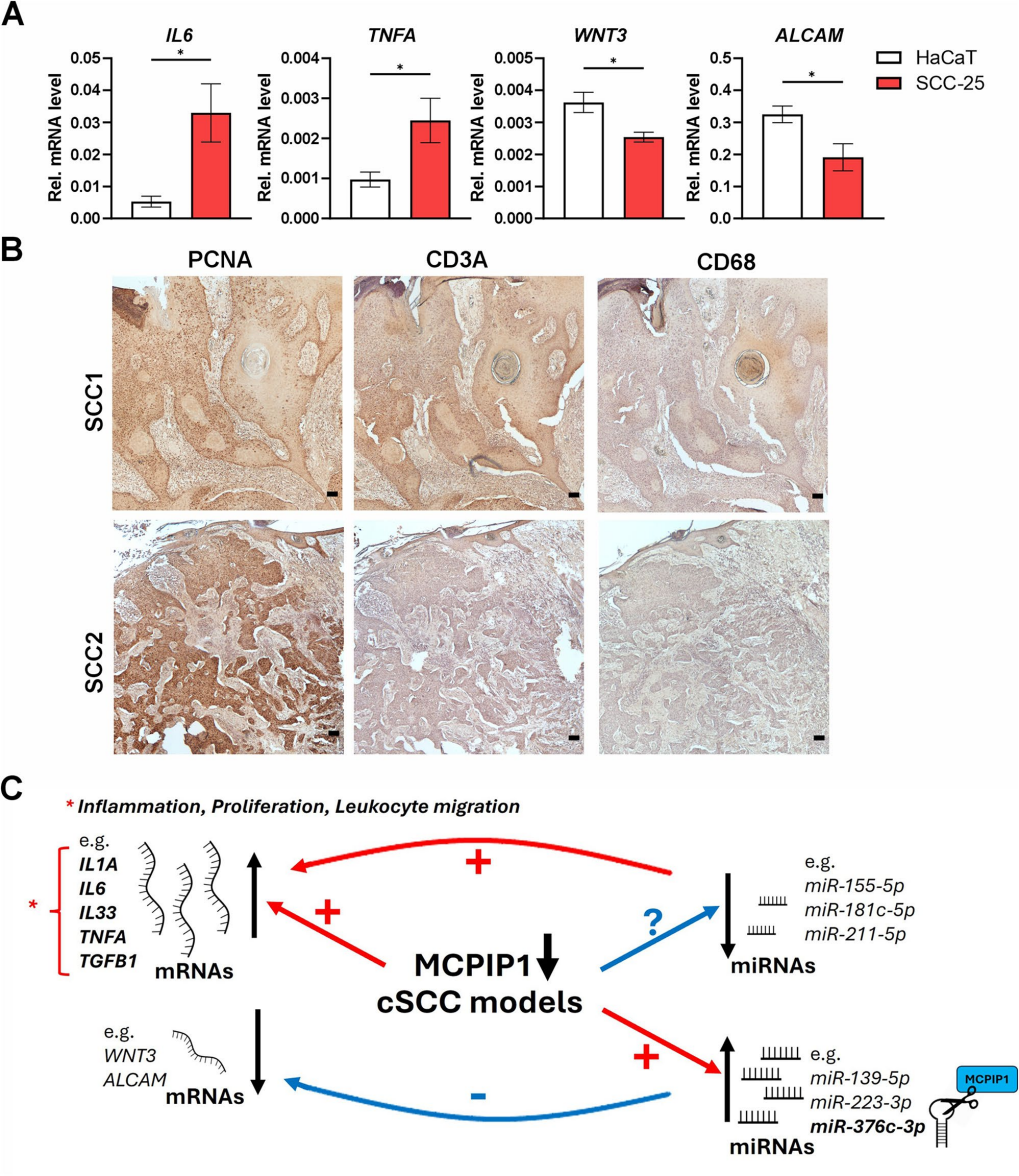
Expression pattern of developmental, proliferation and immune cell markers in SCC models. (**A**) RT‒qPCR analysis of *IL6*, *TNFA*, *WNT3* and *ALCAM* expression in HaCaT and SCC-25 keratinocytes (*n* = 5). *EF2* was used as a reference gene. * – *P* < 0.05 by Student’s t test. (**B**) Representative results of IHC staining for PCNA, CD3A and CD68 markers in cutaneous SCC specimens (*n* = 6). Scale bar 100 μm. (**C**) Graphical summary of the results

In agreement with those predictions, we found that the transcript levels of *IL6* and *TNFA* were significantly higher in malignant (SCC-25) compared to the noncancerous (HaCaT) keratinocytes (Fig. 8A). On the other hand, SCC-25 keratinocytes were characterized by significantly reduced expression levels of genes related to the regulation of developmental processes, *WNT3* and *ALCAM* (Fig. 8A), which correlated with the increased expression of miR-223-3p and miR-376c-3p in these cells (Fig. 3B). In the proposed model, upregulation of *IL6* and *TNFA*, among many other mRNA targets of downregulated miRNAs (Fig. 7A), would affect cancer cell proliferation and migration of leukocytes. We previously demonstrated that in cutaneous SCC, the overall expression of MCPIP1 is reduced and mostly restricted to the SCC-specific structures composed of highly differentiated keratinocytes, keratin pearls. We also showed that regions with high MCPIP1 immunoreactivity generally did not overlap with cells positive for keratin 14, indicating proliferating keratinocytes [8]. As a part of this study, we extended these analyses by investigating the distribution of actively proliferating cells using PCNA marker in six SCC specimens. We also analysed the presence of tumor-infiltrating immune cells: T cells (CD3A+) and macrophages (CD68+). The observed high immunoreactivity for both PCNA and CD3A markers within SCC tumor mass is in agreement with our bioinformatic predictions. Accordingly, our results indicated that all the analyzed specimens showed overall low immunoreactivity for the CD68 + cells. (Fig. 8B).

## Discussion

MCPIP1 exhibits tumor-suppressive properties in many types of cancer (reviewed in [41]), including cSCC [8]. Using a mouse model of chemically induced skin tumorigenesis, we and others recently showed that mice with conditional deletion of MCPIP1 in keratinocytes (Mcpip1^eKO^ mice) develop more aggressive papillomas with faster local growth and increased incidence [8, 42]. To date, the underlying mechanism has been explained most commonly by the dysregulated expression of a plethora of mRNAs; however, MCPIP1 also regulates the levels of miRNAs directly *via* the degradation of pre-miRNAs [18] — and indirectly through the regulation of key modulators of miRNA expression. MiRNAs modulate numerous biological processes, and dysregulation of miRNAs has also been associated with both the promotion and suppression of tumorigenesis, indicating that miRNAs may function as either tumor-specific oncogenes or tumor suppressors [43, 44].

To address the impact of MCPIP1-dependent changes in miRNA profiles during skin carcinogenesis, we used a mouse model of chemically induced skin tumorigenesis with conditional deletion of MCPIP1 in keratinocytes and analyzed miRNA expression in papillomas. In particular, we identified a panel of DE-miRNAs between chemically induced skin papillomas from Mcpip1^eKO^ mice and those from control mice.

More miRNAs were upregulated than downregulated in the Mcpip1^eKO^ group. Among the 18 significantly upregulated DE-miRNAs, the biogenesis of miR-7, miR-21, and miR-146a was previously shown by Suzuki et al. to be directly regulated by MCPIP1 [18]. Using a human epidermal carcinoma in vitro model with downregulation of MCPIP1, we confirmed that the biogenesis of miR-139-5p, miR-223-3p, and miR-376c-3p is also negatively regulated by human MCPIP1. We proved that MCPIP1 directly regulates the expression of miR-376c-3p *via* direct cleave of the corresponding precursor miRNA. In case of miR-223-3p and miR-139-5p, the regulation is most likely indirect. The results of our assays suggested that the mechanism does not include a direct nucleolytic cleavage of pre-223 and pre-139-miRNAs by MCPIP1. However, our experiments were performed in vitro, in non-cancerous environment. It is possible that in the tumor microenvironment, the specificity of MCPIP1 towards its substrate pre-miRNAs is modulated. This may be for example related to the posttranslational modifications of MCPIP1, as we also previously demonstrated [8]. On the other hand, our results showed that the upregulation of miR-92b-3p and miR-196a-5p in Mcpip1^eKO^ papillomas is not a direct consequence of human MCPIP1 enzymatic activity, as the levels of these miRNAs were not increased in human keratinocytes expressing a shRNA targeting MCPIP1. Thus, the plausible origin cells of these miRNAs include both keratinocytes that have been activated by the tumor microenvironment and other cell types present in the tumor mass, such as cancer-associated fibroblasts, T cells, B cells, mast cells and macrophages. Individual miRNAs may further function as regulators of gene expression either intercellularly or in distant cells. For example, miR-92b, which is the most significantly upregulated miRNA in Mcpip1^eKO^ papillomas according to our miRNA-Seq data (FC = 3.3), has been shown to promote immunosuppressive macrophage polarization [45]. Moreover, it has been described as a potent oncogene (oncomiR) that promotes cell proliferation and invasion in several types of tumors, including non-small cell lung cancer (NSCLC) and esophageal SCC [46, 47]. Thus, we hypothesize that miR-92b-3p is an important oncogenic driver also in cSCC, although it is not directly regulated by keratinocyte MCPIP1.

On the other hand, in our model of skin papillomagenesis, loss of MCPIP1 in keratinocytes resulted in reduced expression of 15 miRNAs. The most significantly altered miRNA, miR-211-5p (FC = 3.2), has been previously described as an inhibitor of the invasive properties of many types of cancers [48, 49], including SCC [50]. The mechanism explaining how downregulation of MCPIP1 leads to the downregulation of a plethora of miRNAs in our model remains however elusive. We previously demonstrated that downregulation of MCPIP1 shapes skin tumor environment towards more aggressive phenotype. This may affect several pathways, i.e. related to the alteration of genomic miRNA copy numbers or alterations in the availability of transcription factors, which may result in the suppression of certain miRNAs.

To investigate the relevance of the shortlisted SCC keratinocyte-derived miRNAs whose levels were negatively correlated with the activity of MCPIP1, we compared their relative expression between human noncancerous keratinocytes (HaCaT cells) and malignant human keratinocytes (SCC-25 cells). The expression of miR-139-5p, miR-223-3p, and miR-376c-3p was significantly greater in SCC-25 cells than in HaCaT cells, suggesting the potential involvement of these miRNAs as oncomiRs in the development or progression of cSCC. On the other hand, analysis of miRNA expression profiles from cutaneous BCCs (cBCCs) and normal epithelial skin revealed upregulation of miR-223-3p but not miR-376c-3p and miR-139-5p in cancerous tissues. These results indicate the existence of diverse miRNA-driven underlying mechanisms during cBCC and cSCC.

Individual miRNAs may act as either tumor promoters or suppressors, depending on the cellular or tissue context [43]. This observation is also true for miR-223. Previously published data indicate that the activity of miR-223 is related to different inflammatory disorders and cancer, but miR-223 may act as either an oncomiR or a tumor suppressor in a context-dependent manner [51, 52]. Similarly, miR-376c-3p has been described to function as a tumor promoter in hepatocellular and gastric carcinomas [53, 54]. However, it has been proposed to exert tumor-suppressive effects in neuroblastoma [55]. Overall, the functions of selected miRNAs in cSCC remain unknown. We addressed this knowledge gap by analyzing the consequences of ectopic overexpression of miR-223-3p, miR-376c-3p, and miR-139-5p *via* specific miRNA mimics. Overexpression of miR-223-3p, miR-376c-3p, and, to a lesser extent, miR-139-5p accelerated the cell cycle compared to that in the corresponding NC mimic groups; this acceleration was shown quantitatively as a significant shift in the cell cycle distribution from the resting G0/G1 phase to the proliferating S and G2/M phases. At the molecular level, this effect was accompanied by increased expression of the main cyclins (Cyclin D1 and Cyclin B1) and other key cell cycle modulators (CDK4, Wee1, and Myt1), whose overexpression is a hallmark of malignant cells [56–58]. Furthermore, our results imply that in SCC, upregulation of miR-376c-3p is an important step driving epithelial to mesenchymal transition.

We next integrated experimental and predictive data on the mRNA interactions of all miRNAs altered in Mcpip1^eKO^ papillomas using the TarBase v7 database and DIANA-microT-CDS algorithm. Comparisons between the two sets of genes indicated that in each condition, approximately half of the experimentally validated miRNA target genes (data obtained from TarBase v7) were also annotated in MicroT-CDS. Overall, our results revealed that ~ 35% of the DE-PCGs (FC > 1.6 and *P* adj. < 0.05) contained at least one DE-miRNA target site. It was previously speculated using computational predictions that a single miRNA can modulate the level of hundreds of coding RNAs in the genome [44]. As we expected, the number of putative interactions was greater than the number of shortlisted unique genes, indicating that a single gene is a putative target of multiple DE-miRNAs also in our model. To identify novel miRNA‒mRNA interactions in the environment of cutaneous malignancy in humans and considering that 3’UTR sequences are less conserved than CDSs, the murine gene symbols were converted into those of their human miRNA and mRNA orthologs. Bioinformatic analyses indicated that nearly 70% of the miRNA target genes in mice were conserved in humans.

To identify novel miRNA-driven mechanisms based on our experimental model and computational predictions, functional analyses were performed on the sets of human miRNA‒mRNA interactions predicted by microT-CDS and TarBase v7. The largest number of upregulated miRNA target genes were functionally annotated to the Wnt signaling pathway and the regulation of cell development. We also shortlisted genes related to the cellular response to BMP stimulation. Deregulated Wnt signaling has been implicated in many types of cancer, in which it leads to abnormal proliferation and differentiation of both stem cells and progenitors [59]. Among the functionally upregulated miRNA target genes were prominent tumor suppressors, such as TP53INP1/2, which encodes a stress-induced p53 target gene, and the adhesion molecule CDH10 [60]. The putative targets of miR-223-3p included the gene encoding ELF-5, which has been reported to possess tumor-suppressive properties in many types of cancer by inhibiting cancer cell proliferation, migration and invasion [61]. *TRIM2*, a shortlisted putative target of miR-376c-3p, and *BNC2* (a putative common target of both miR-223-3p and miR-376c-3p) were also previously described as candidate antitumor genes [62]. Thus, we hypothesized that downregulated expression of these factors is a likely contributor to the hyperproliferative phenotype of keratinocytes overexpressing miR-223-3p and miR-376c-3p in both murine and human models.

From the bioinformatic analyses, we concluded that in both analyses of miRNA interactions in both humans and mice, more genes were identified as targets of the 15 downregulated miRNAs than of the 18 upregulated miRNAs, suggesting the greater functional relevance of the downregulated miRNAs implicated in cSCC carcinogenesis. Most of the shortlisted target genes of the downregulated miRNAs were related to inflammatory processes, regulation of cell‒cell adhesion, and epithelial cell proliferation.

We previously demonstrated that MCPIP1 affects skin cancer progression by regulating the expression of inflammatory molecules such as IL-6, IL-33 and TGF-β [8]. The mechanism includes direct degradation of corresponding transcripts or indirectly by controlling the level of key transcriptional regulators. This study adds towards molecular understanding of the mechanism of MCPIP1 activity in skin carcinogenesis *via* addressing the changes of the miRNA profiles. Our analyses revealed that the greatest impact is related to the upregulated expression of a plethora of inflammatory factors like IL-6, IL1-a, or TNF-a, which are the target genes of the downregulated miRNAs. In the proposed mechanism, their deregulated expression in SCC would enhance inflammation and epithelial proliferation. Accordingly, our IHC analyses of cSCC specimens indicated increased immunoreactivity towards proliferation marker (PCNA) and leukocyte marker (CD3), suggesting important role for MCPIP1 in the regulation of epithelial proliferation and migration of lymphocytes. Based on our findings, these processes are at least partially regulated or enhanced by the MCPIP-mediated miRNA-mRNA network (Fig. 8C).

In our datasets, numerous pro-oncogenic factors were identified; these included the protooncogene SPI1; SERPINE1, a biomarker of SCC progression; and the matrix metalloproteinase MMP-13. Interestingly, Gene Ontology enrichment analysis revealed a group of genes among which most encoded essential transcription factors, including KLF5, ETS-1, SPI-1, and c-Fos, as modulators of miRNA transcription. In the proposed mechanism, miRNA-mediated upregulation of target mRNAs modulates the miRNA landscape *via* a feedback loop. The exact mechanism is currently being investigated.

In conclusion, in the current study, we demonstrated profound changes in miRNA profiles in skin malignancies promoted by the loss of MCPIP1. The associated miRNA‒mRNA interplay shapes the balance between pro- and antitumorigenic signaling and seems to play a critical role in the progression of tumors. In addition, miRNAs modulate cellular signaling pathways at multiple levels, i.e., *via* interactions with coding and noncoding RNA molecules and through the repression of protein translation. The miRNA-mediated effects on the noncoding transcriptome is the subject of our follow-up study.

## Conclusions

In summary, our study describes the changes in the expression patterns of miRNAs and their target genes caused by downregulation of MCPIP1 in SCC. This study also provides a list of novel potential biomarkers that can be investigated as early diagnostic markers or therapeutic targets in human SCC. An understanding of the key molecular factors involved in early stages of carcinogenesis is crucial for the diagnosis and for the development of novel targeted therapies.

## Electronic supplementary material

Below is the link to the electronic supplementary material.

Supplementary Material 1: Additional file 6 - Table S3. Interactions between human orthologs of the upregulated in Mcpip1eKO papillomas miRNAs and downregulated DE-PCGs identified using DIANA-microT-CDS. Additional file 7 - Table S4. Interactions between human orthologs of the upregulated in Mcpip1eKO papillomas miRNAs and downregulated DE-PCGs identified using DIANA-TarBase. Additional file 8 - Table S5. Interactions between human orthologs of the downregulated in Mcpip1^eKO^ papillomas miRNAs and upregulated DE-PCGs identified using DIANA-microT-CDS. Additional file 9 - Table S6. Interactions between human orthologs of the downregulated in Mcpip1eKO papillomas miRNAs and upregulated DE-PCGs identified using DIANA-TarBase.

Supplementary Material 2: Additional file 1 - Table S1. List of primer sequences used in this study.

Supplementary Material 3: Additional file 2 - Figure S1. Heatmap showing the expression levels of selected DE-PCGs in Mcpip1eKO papillomas.

Supplementary Material 4: Additional file 3 – Table S2. List of upregulated in Mcpip1^eKO^ papillomas pre-miRNAs.

Supplementary Material 5: Additional file 4 - Figure S2. Expression levels of selected miRNAs in malignant keratinocytes and regulation by MCPIP1.

Supplementary Material 6: Additional file 5 - Figure S3. Increased expression of epithelial to mesenchymal transition activating factors in SCC-25 cells overexpressing miR-376c-3p mimic.

## Data Availability

Sequencing data are available at NCBI BioProject database under PRJNA1076100. Other data sets generated during the current study are available from the corresponding author on reasonable request.
