# Supplementary material for: MCPIP1 modulates the miRNA‒mRNA landscape in keratinocyte carcinomas
Source: J Exp Clin Cancer Res. 2024 Oct 21;43:290. doi: 10.1186/s13046-024-03211-8 (PMC11492624; doi:10.1186/s13046-024-03211-8)
Supplement: Supplementary file 2 — Supplementary Material 2: Additional file 1 - Table S1. List of primer sequences used in this study. [file 13046_2024_3211_MOESM2_ESM.docx]

**Additional file 1**

**Table S1.** List of primer sequences used in this study.

|  |  | **For (5’->3’)** | **Rev (5’->3’)** |
| --- | --- | --- | --- |
| **Primes used for RT-qPCR** | ***ALCAM*** | GACAACGTGTTTGAGGCACC | TCTTCTGAAATGCAGTCACCCA |
|  | ***CTNNB1*** | AAAATGGCAGTGCGTTTAG | TTTGAAGGCAGTCTGTCGTA |
|  | ***EF2*** | GACATCACCAAGGGTGTGCAG | TCAGCACACTGGCATAGAGGC |
|  | ***IL6*** | GTGAAAGCAGCAAAGAGGCA | TCACCAGGCAAGTCTCCTCA |
|  | ***SNAI1*** | CTCTAATCCAGAGTTTACCTTC | GACAGAGTCCCAGATGAG |
|  | ***TNFA*** | CAGGCGGTGCTTGTTCCTCAG | GGGCTACAGGCTTGTCACTCG |
|  | ***TRIM2*** | CCAACAGGTGTAGCAGTGGA | CCACTCCCATCAAAAACCTGG |
|  | ***VIM*** | TCTACGAGGAGGAGATGCGG | GGTCAAGACGTGCCAGAGAC |
|  | ***WNT3*** | ACTATTGGGGGCGTCGCT | GAGGCCAGAGATGTGTACTGC |
|  | ***Zc3h12a*** | CAGCCTCGACCAGATGTGCC | CAGCCGCTCCTCGATGAAGC |
| **Primers used for cloning** | **hsa-pre--miR-223** | GATCCCGTGTATTTGACAAGCTGAGTTGGACACTCCATGTGGTAGAGTGTCAGTTTGTCAAATACCCCAG | AATTCTGGGGTATTTGACAAACTGACACTCTACCACATGGAGTGTCCAACTCAGCTTGTCAAATACACGG |
|  | **hsa-pre-miR-376c** | GATCCGGTGGATATTCCTTCTATGTTTATGTTATTTATGGTTAAACATAGAGGAAATTCCACGTG | AATTCACGTGGAATTTCCTCTATGTTTAACCATAAATAACATAAACATAGAAGGAATATCCACCG |
|  | **hsa-pre-miR-139** | GATCCTCTACAGTGCACGTGTCTCCAGTGTGGCTCGGAGGCTGGAGACGCGGCCCTGTTGGAGG | AATTCCTCCAACAGGGCCGCGTCTCCAGCCTCCGAGCCACACTGGAGACACGTGCACTGTAGAG |
