# Supplementary material for: MCPIP1 modulates the miRNA‒mRNA landscape in keratinocyte carcinomas
Source: J Exp Clin Cancer Res. 2024 Oct 21;43:290. doi: 10.1186/s13046-024-03211-8 (PMC11492624; doi:10.1186/s13046-024-03211-8)
Supplement: Supplementary file 3 — Supplementary Material 3: Additional file 2 - Figure S1. Heatmap showing the expression levels of selected DE-PCGs in Mcpip1eKO papillomas. [file 13046_2024_3211_MOESM3_ESM.docx]

**Additional file 2 - Figure S1**


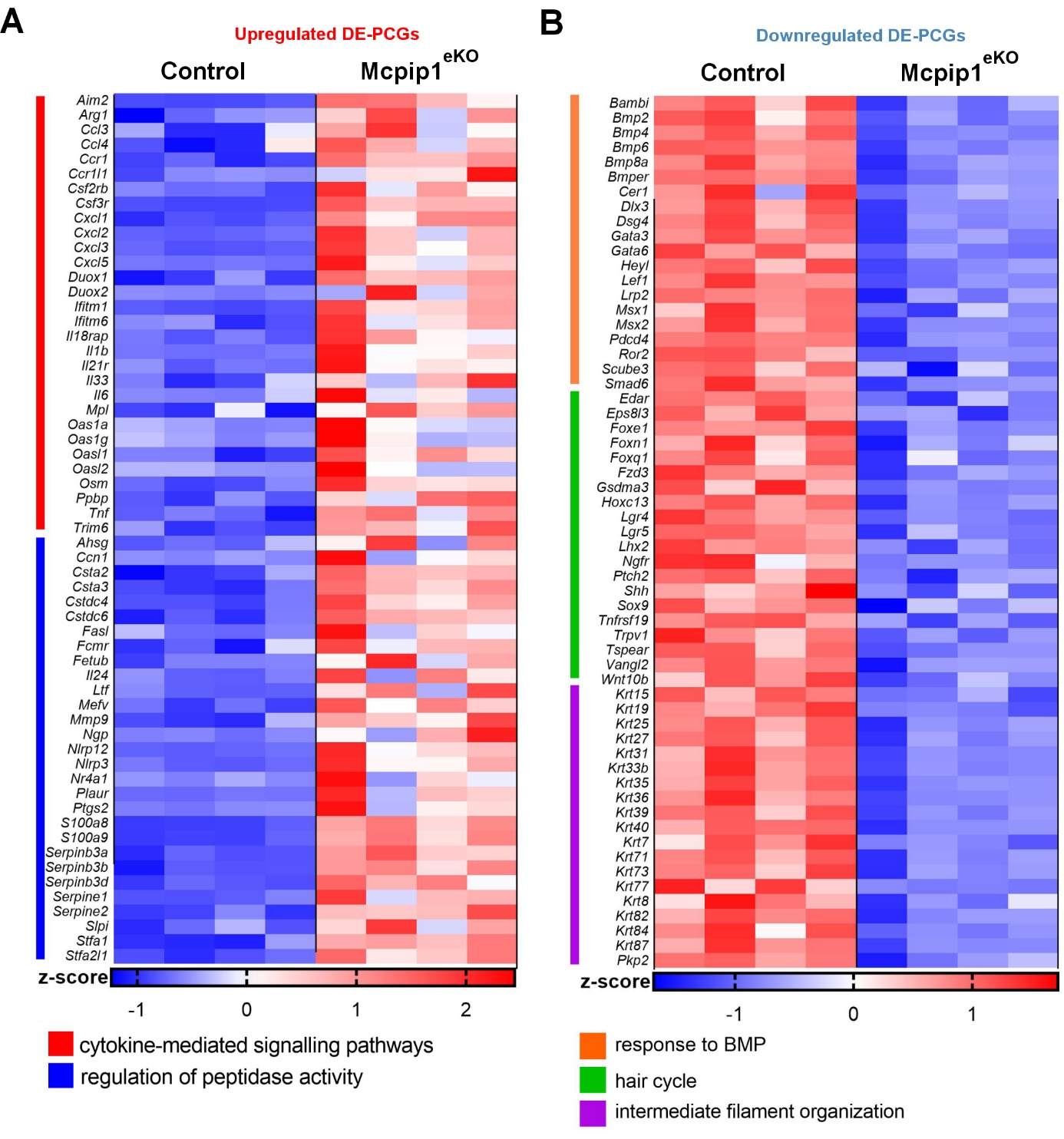


**Figure S1. Heatmap showing the expression levels of selected DE-PCGs in Mcpip1^eKO^ papillomas. A.** Heatmap showing the expression levels (z scores) of representative DE-PCGs related to the selected upregulated in Mcpip1^eKO^ papillomas GO terms**. B.** Heatmap showing the expression levels (z scores) of representative DE-PCGs related to the selected downregulated in Mcpip1^eKO^ papillomas GO terms**.** The top 20-30 DE-PCGs (based on FC scores) from each GO term is shown. DE, differentially expressed.
