# Supplementary material for: MCPIP1 modulates the miRNA‒mRNA landscape in keratinocyte carcinomas
Source: J Exp Clin Cancer Res. 2024 Oct 21;43:290. doi: 10.1186/s13046-024-03211-8 (PMC11492624; doi:10.1186/s13046-024-03211-8)
Supplement: Supplementary file 4 — Supplementary Material 4: Additional file 3 – Table S2. List of upregulated in Mcpip1eKO papillomas pre-miRNAs. [file 13046_2024_3211_MOESM4_ESM.docx]

**Additional file 3**

**Table S2.** List of upregulated in Mcpip1^eKO^ papillomas pre-miRNAs.

| **mirna** | **MirBase_ID** | **MirGeneDB_ID** | **FC** | **padj** |
| --- | --- | --- | --- | --- |
| mmu-mir-92b | MI0005521 | Mmu-Mir-92-P1d_pre | 3,306984 | 0,002488 |
| mmu-mir-7007 | MI0022856 |  | 2,895171 | 0,028322 |
| mmu-mir-223 | MI0000703 | Mmu-Mir-223_pre | 2,477842 | 1,46E-07 |
| mmu-mir-144 | MI0000168 | Mmu-Mir-144-v1_pre | 2,362154 | 0,00862 |
| mmu-mir-146b | MI0004665 | Mmu-Mir-146-P1_pre | 2,34069 | 1,56E-13 |
| mmu-mir-7a-1 | MI0000728 | Mmu-Mir-7-P2_pre | 2,16768 | 2,46E-13 |
| mmu-mir-7a-2 | MI0000729 | Mmu-Mir-7-P1_pre | 2,107727 | 1,35E-10 |
| mmu-mir-376c | MI0003533 | Mmu-Mir-376-P4_pre | 2,050951 | 0,024402 |
| mmu-mir-21a | MI0000569 | Mmu-Mir-21_pre | 1,982491 | 3,51E-08 |
| mmu-mir-146a | MI0000170 | Mmu-Mir-146-P4_pre | 1,884234 | 6,85E-10 |
| mmu-mir-139 | MI0000693 | Mmu-Mir-139_pre | 1,837905 | 0,000759 |
| mmu-mir-196a-2 | MI0000553 | Mmu-Mir-196-P4_pre | 1,768876 | 0,011211 |
| mmu-mir-455 | MI0004679 | Mmu-Mir-455_pre | 1,751927 | 0,035559 |
| mmu-mir-196a-1 | MI0000552 | Mmu-Mir-196-P3_pre | 1,68141 | 0,028322 |
| mmu-mir-214 | MI0000698 | Mmu-Mir-214-v1_pre | 1,640827 | 0,01107 |
| mmu-mir-379 | MI0000796 | Mmu-Mir-154-P7_pre | 1,613588 | 0,014437 |
