# Supplementary material for: MCPIP1 modulates the miRNA‒mRNA landscape in keratinocyte carcinomas
Source: J Exp Clin Cancer Res. 2024 Oct 21;43:290. doi: 10.1186/s13046-024-03211-8 (PMC11492624; doi:10.1186/s13046-024-03211-8)
Supplement: Supplementary file 5 — Supplementary Material 5: Additional file 4 - Figure S2. Expression levels of selected miRNAs in malignant keratinocytes and regulation by MCPIP1. [file 13046_2024_3211_MOESM5_ESM.docx]

**Additional file 4 - Figure S2**


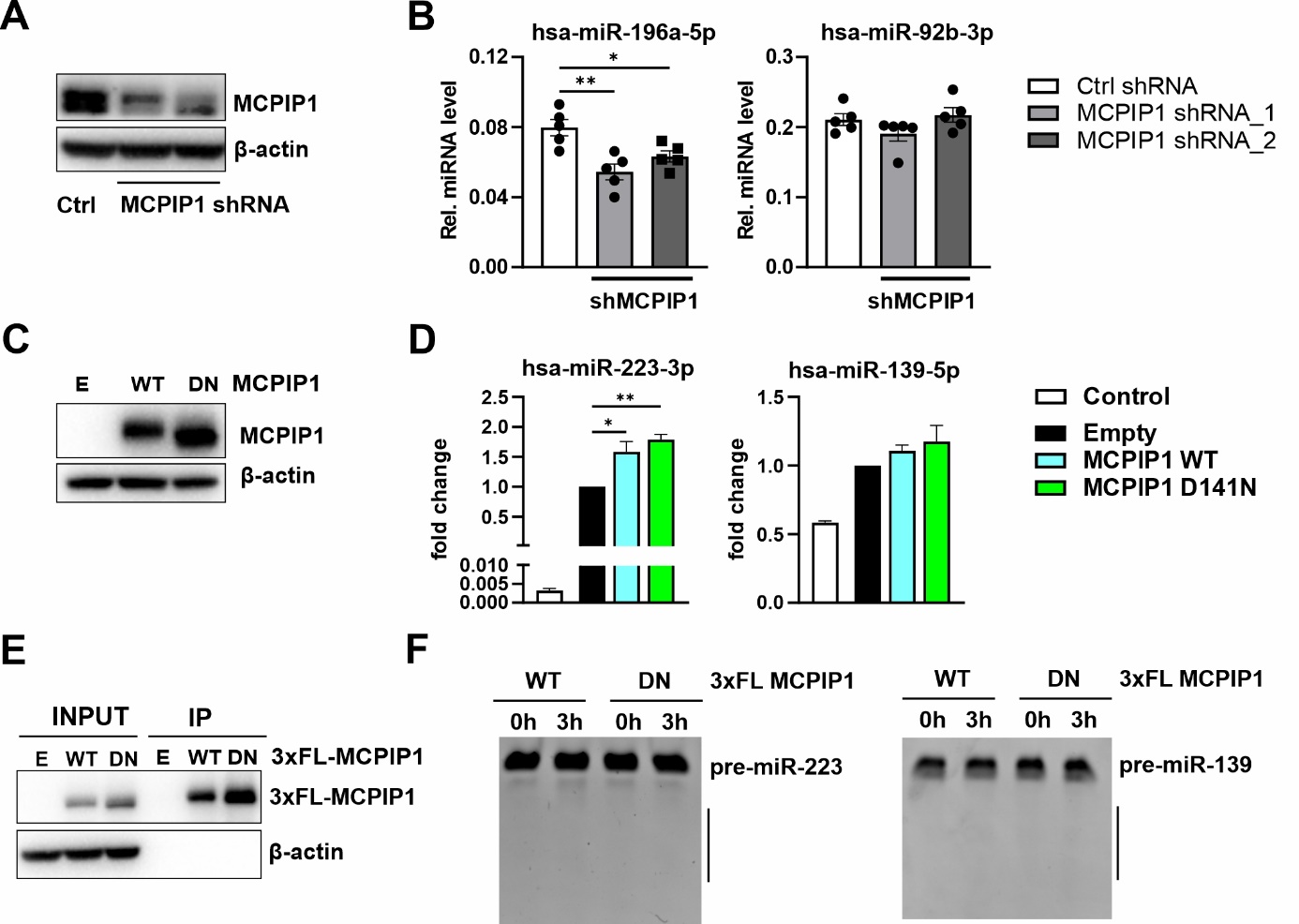


**Figure S2. Expression levels of selected miRNAs in malignant keratinocytes and regulation by MCPIP1. A.** Western blot for MCPIP1 and β-actin expression in A431 cell lysates stably expressing control or MCPIP1-specific shRNA. **B.** RT-qPCR analysis of hsa-miR-196a-5p and hsa-miR-92b-3p in A431 cells expressing the control (NC, negative control) or MCPIP1-specific shRNA (n=4-5). **C.** Western blot for MCPIP1 and β-actin in HEK293 cells transfected with pcDNA3.0 (empty), MCPIP1-WT or MCPIP1-D141N. **D.** RT-qPCR analysis of miRNA expression in HEK293 cells co-transfected with pcDNA3.0, MCPIP1-WT or MCPIP1-D141N with pcDNA3.0 expressing pre-miR-223 or pre-miR-139 (n=3). **E.** Western blot for FLAG and β-actin in input and protein lysates following IP with anti-FLAG from HaCaT cells expressing 3xFLAG-MCPIP1-WT or 3xFLAG-MCPIP1-D141N. **F.** Image of RNA electrophoresis in 8% PAA gel indicating results of degradation assays using 3xFLAG MCPIP1 or 3xFLAG MCPIP1-D141N immunoprecipitated from HaCaT cells with *in vitro* transcribed pre-miR-223 or pre-miR-139 as a substrate. * – *P* < 0.05; ** – *P*<0.01 by Student’s t test *t*-test. Hsa, *Homo sapiens*.
