# Supplementary material for: MCPIP1 modulates the miRNA‒mRNA landscape in keratinocyte carcinomas
Source: J Exp Clin Cancer Res. 2024 Oct 21;43:290. doi: 10.1186/s13046-024-03211-8 (PMC11492624; doi:10.1186/s13046-024-03211-8)
Supplement: Supplementary file 6 — Supplementary Material 6: Additional file 5 - Figure S3. Increased expression of epithelial to mesenchymal transition activating factors in SCC-25 cells overexpressing miR-376c-3p mimic. [file 13046_2024_3211_MOESM6_ESM.docx]

**Additional file 5 - Figure S3**


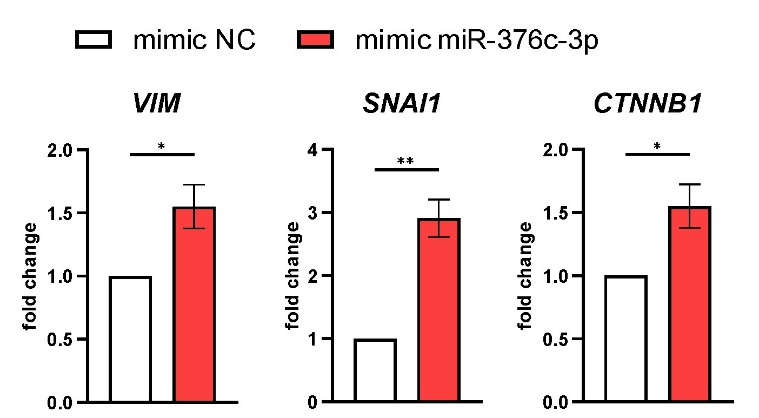


**Figure S3. Increased expression of epithelial to mesenchymal transition activating  factors in SCC-25 cells overexpressing miR-376c-3p mimic.** RT-qPCR analysis of *VIM*, *SNAI1*, and *CTNBB1* in SCC-25 cells transfected with 25nM of the control or miR-376c-3p mimic for 72h (n=3). *EF2* was used as a reference gene. * – *P* < 0.05; ** – *P*<0.01 by Student’s t test *t*-test. NC, negative control.
